# Advances in Mammalian Cell Line Development Technologies for Recombinant Protein Production

**DOI:** 10.3390/ph6050579

**Published:** 2013-04-26

**Authors:** Tingfeng Lai, Yuansheng Yang, Say Kong Ng

**Affiliations:** 1 Bioprocessing Technology Institute, Agency for Science, Technology and Research (A*STAR), 20 Biopolis Way, #06-01, Centros, 138668, Singapore; E-Mails: ethan_lai@bti.a-star.edu.sg (T.L.); yang_yuansheng@bti.a-star.edu.sg (Y.Y.); 2 School of Chemical and Biomedical Engineering, Nanyang Technological University, N1.2-B2-33, 62 Nanyang Avenue, 637459, Singapore; 3 Department of Pharmacy, Faculty of Science, National University of Singapore, Singapore

**Keywords:** cell line development, protein expression, clone screening, biopharmaceutical production

## Abstract

From 2006 to 2011, an average of 15 novel recombinant protein therapeutics have been approved by US Food and Drug Administration (FDA) annually. In addition, the expiration of blockbuster biologics has also spurred the emergence of biosimilars. The increasing numbers of innovator biologic products and biosimilars have thus fuelled the demand of production cell lines with high productivity. Currently, mammalian cell line development technologies used by most biopharmaceutical companies are based on either the methotrexate (MTX) amplification technology or the glutamine synthetase (GS) system. With both systems, the cell clones obtained are highly heterogeneous, as a result of random genome integration by the gene of interest and the gene amplification process. Consequently, large numbers of cell clones have to be screened to identify rare stable high producer cell clones. As such, the cell line development process typically requires 6 to 12 months and is a time, capital and labour intensive process. This article reviews established advances in protein expression and clone screening which are the core technologies in mammalian cell line development. Advancements in these component technologies are vital to improve the speed and efficiency of generating robust and highly productive cell line for large scale production of protein therapeutics.

## 1. Introduction

The approval of Chinese hamster ovary (CHO)-derived tissue plasminogen activator (tPA, Activase) in 1986 revolutionized medicine and raised the possibility of using mammalian cell culture for the manufacturing of protein therapeutic products. More than 20 years after tPA approval, CHO cells remained as the preferred mammalian cell line for the production of recombinant protein therapeutic for several reasons. First, CHO cells are capable of adapting and growing in suspension culture which is ideal for large scale culture in the industry. Second, CHO cells pose less risk as few human viruses are able to propagate in them [1]. Third, CHO cells can grow in serum-free and chemically defined media which ensures reproducibility between different batches of cell culture. Fourth, CHO cells allow post translational modifications to recombinant proteins which are compatible and bioactive in humans [2]. Specifically, glycosylation of glycoproteins produced by CHO cells are more human-like, with the absence of immunogenic α-galactose epitope [3]. Fifth, several gene amplification systems are well established to make use of the genome instability of CHO cells to allow for gene amplification which ultimately result in higher yield of recombinant protein. Currently, recombinant protein titers from CHO cell culture have reached the gram per liter range which is a 100-fold improvement over similar process in the 1980s. The significant improvement of titer can be attributed to progress in establishment of stable and high producing clones as well as optimization of culture process. Due to these reasons, CHO cells are established host cell lines for regulatory approvals of therapeutic glycoprotein products [1,2,4].

Since the first approval and up to 2011, 96 recombinant protein therapeutics produced from mammalian cells have been approved, commanding USD 112.93 billion dollar annual revenue [5]. These numbers continue to grow with the biopharmaceutical industry, which saw an average of 15 new approvals per year by US Food and Drug Administration (FDA) from 2006 to 2011. At the same time, expiration of patent protection that grant exclusive rights to produce blockbuster biologics such as Epogen (erythropoietin) and Remicade (infliximab) has fuelled the demand of biosimilars [6]. A common feature in the development of innovator products and biosimilars is that new production cell lines have to be developed. This involves the selection of stable cell clones with high productivity to be further developed for large scale manufacturing via culture medium and process optimization.

**Figure 1:**
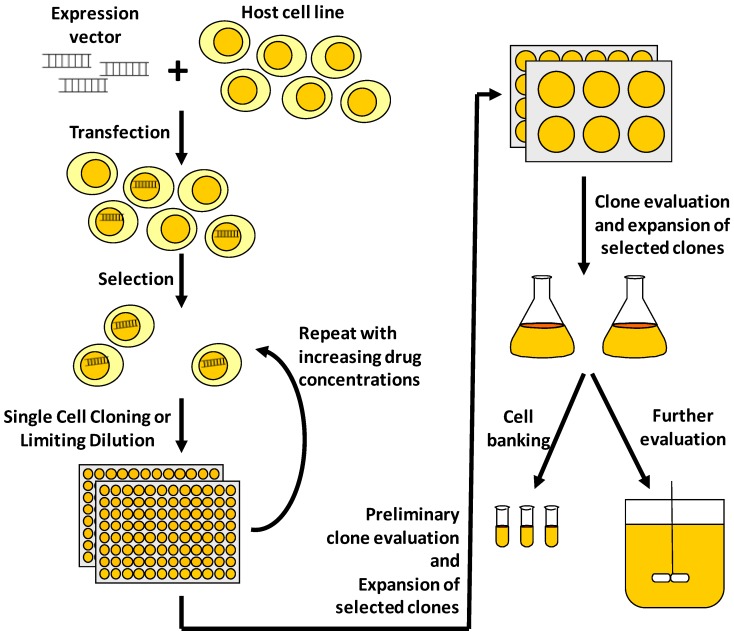
Illustration of a typical process to develop a mammalian cell line for recombinant protein manufacturing. After transfection of the host cell line with the expression vector containing the gene of interest (GOI) and selection marker, the cells undergo drug selection and cloning to derive cells that are producing the GOI. When gene amplification systems are used, concentrations of selection drug (e.g., MTX or MSX) can be increased step-wise to derive cell clones that are more productive. Cell clones with high recombinant protein titer are chosen for progressive expansions before cell banking and further clone evaluations, such as production stability of the cell clones and quality of recombinant protein.

Currently, cell line development technologies used by most biopharmaceutical companies are based on either the methotrexate (MTX) amplification technology that originated from the 1980s [7], or Lonza’s glutamine synthetase (GS) system [8,9,10]. Both systems make use of a specific drug to inhibit a selectable enzyme marker essential for cellular metabolism: MTX inhibits dihydrofolate reductase (DHFR) in the MTX amplification system, and methionine sulphoximine (MSX) inhibits GS in the GS system. Complementary to these drug/enzyme pairs are cell lines that are deficient in these enzymes. While CHO cell lines deficient in DHFR has been established since the 1980s [11,12], that for GS is only developed recently [13,14]. A typical cell line development scheme using these technologies is illustrated in Figure 1. After transfection with expression vectors containing the expression cassettes for the recombinant protein and selection marker genes, the cells are selected and gene-amplified with the selection drug, for example MTX or MSX. Here, gene amplification describes the increase in recombinant gene copy number in the cells [15] commonly associated with, but not limited to, the applications of MTX and MSX. MTX or MSX concentration can also be increased step-wise to further increase cell protein productivity by further gene amplification. Single cell cloning or limiting dilution is then performed to ensure that the selected cells for further processing are producing the recombinant protein. Analyses of protein titers are subsequently used to choose the clones for progressive expansions. Finally, selected clones are evaluated in controlled bioreactors and banked for future use [16,17].

In addition to being a regulatory requirement, single cell cloning or limiting dilution is technically necessary in the process because the protein productivity of individual cells in the transfected and gene amplified cell populations are varied. This heterogeneity of the clones is commonly attributed to the random integration of the transfected gene of interest into the genome [18]. Since the chromosomal surroundings exert strong influences on the promoter which in turn affects the transcription rate of the gene of interest, it is difficult to obtain homogenous level of protein expression among individual transfected cells [19]. Furthermore, the gene amplification process results in large genomic rearrangements that lead to further heterogeneity in protein expression levels [7,20]. In addition to the variation caused by random gene integration and gene amplification, it is found that the genome of the CHO cell population exhibits rapid genetic changes which arise from random mutations and genetic drift. [21]. Furthermore, it is demonstrated that intraclonal protein expression is unexpectedly heterogenous with a standard deviation of 50% to 70% of the mean and they undergo stochastic fluctuations in their expression [22]. Another study has also shown that chromosomal aberrations occur in more than half of the cell lines studied when a recombinant CHO DG44 cell line was established [23]. As a result of the dynamic nature of the CHO cell genome, clonal derivatives, deviating from the original parental population, have displayed heritable variance in attributes like specific proliferation rate and *N*-glycan processing of expressed recombinant protein [21]. Taken together, even though the clonal CHO cell population may be varied due to inherent genomic instability, the heterogeneity caused by random gene integration and gene amplification can be alleviated by cloning, and this can contribute to a more consistent bioreactor performance, product quality and manufacturing process.

Another reason for cloning the cells is that high producing clones occur rarely in the heterogeneous cell populations after transfection and gene amplification [24,25,26,27,28]. Hence, in situations where high recombinant protein productivity is essential for product commercial viability (for example, the production of monoclonal antibody therapeutics) thousands of clones have to be screened to obtain a set of production clones. While the screening process can be assisted with modern technologies and robotics, the production cell line development process is still a time, labour and capital intensive endeavour, that typically requires 6 to 12 months.

Advances in cell line development technologies are therefore crucial to support the rapid development of recombinant protein therapeutic products, where improvements in the timeline and the ease of generating high producing cell lines in an academic setting can contribute to the faster development of biosimilars and innovator products alike. In the case of innovator products, a reduction in time-to-market period for biopharmaceutical manufacturers is also advantageous because it maximizes profit for biologics with the limited period of patent exclusivity. Advances in cell line development technologies centre on improvements in protein expression technologies and new clone screening technologies. In this review, we attempt to summarize established advances in these component technologies that can lead to the development of robust and highly productive cell lines for large-scale commercial production of protein therapeutics.

## 2. Protein Expression Technologies

**Figure 2:**
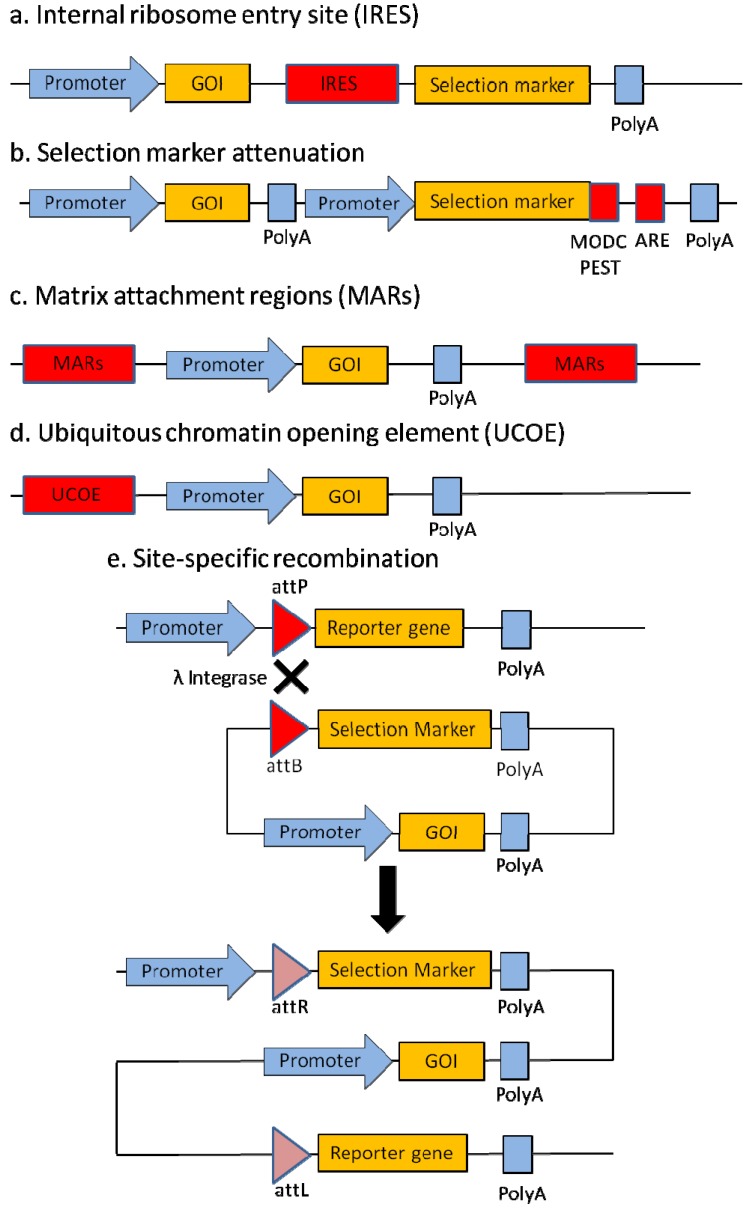
Illustration of expression vector engineering technologies for recombinant protein production in mammalian cells. (**a**) Selection marker attenuation increases the selection stringency which leads to increased probability of isolating cell clones with high productivity of the recombinant gene of interest (GOI). One of the strategies for selection marker attenuation involves the use of mRNA and protein destabilizing elements such as AU-rich elements (ARE) and murine ornithine decarboxylase (MODC) PEST region respectively to reduce the expression of the selection marker. (**b**) The internal ribosome entry site (IRES) is used to link expression of multiple genes. Placement of the selection marker gene downstream of the gene of interest ensures that expression of selection marker is dependent on the successful transcription of the GOI. (**c**) Matrix attachment regions (MARs) flanking the gene of interest promote gene expression by creation of chromatin loops, which maintain a transcriptionally active chromatin structure. (**d**) Ubiquitous chromatin opening elements (UCOEs) flanking the gene of interest augment gene expression by sustaining the chromatin in an “open” configuration. (**e**) Example of site-specific recombination illustrated here uses mutant λ integrase to integrate an expression cassette into the genome. The recombination event is irreversible as attP and attB target sequences are changed to attR and attL sites upon recombination. A successful recombination event will also place the promoterless selection marker gene downstream of a promoter and activate its expression to facilitate cell clone selection, while disrupting the expression of the reporter gene. Site-specific recombination to integrate the GOI into a previously determined genomic hotspot is thus promoted with the use of the recombinase and its corresponding target sequences. Identification of such genomic hotspot is dependent on the expression level of a single copy of reporter gene randomly integrated into the genome.

Productivity of cell culture titer can be increased through the modulation of transcriptional activity via expression vector engineering (Figure 2) by modulating the co-expression of product and selection marker genes, the stringency of the selection marker, the DNA regulatory elements carried on the vector, and targeting its integration site on the host cell genome. Productivity can also be increased through improving cell culture characteristics via cell line engineering. In this section, different protein expression technologies that facilitate the establishment of stable high producing cell lines will be discussed.

### 2.1. Internal Ribosome Entry Site (IRES)

Product and selection genes can be co-expressed by co-transfection of mammalian cells with separate vectors. The strategy is limited by the inefficiency of co-transfection and the reliability of product expression based on the selection of the cotransfected marker gene can be very low [20]. Expression of the product and marker genes on the same vector partially improves the reliability of selection of product expression [29]. However, the use of multiple promoters in one vector may result in transcriptional interference, suppression of one active transcriptional unit on another unit in stable transfections [9].

These problems can be solved with the applications of IRES elements. There are many reported IRES elements which can be broadly categorized into cellular or viral IRESes [30]. Expression of multiple genes such as selection marker and gene of interest can be linked by insertion of an IRES element between the two genes. This allows both genes to be dependent on the same promoter for transcription into a single mRNA. The IRES on the mRNA then allows for the 5' cap-independent translation initiation of the downstream gene, while the transcription initiation of the upstream gene is 5' cap-dependent. Hence, two different proteins can be translated from the single mRNA.

There are several advantages to linking the expression of multiple genes through the use of IRES. First, a single promoter can be used to drive the transcription of the polycistronic mRNA and ensure a more consistent expression ratio of the linked genes [31]. This is demonstrated to be advantageous for the successfully expression of heterodimeric protein like antibody which is dependent on the balanced expression of the heavy and light chains [31,32]. Second, by designing the selection marker gene as the downstream gene in an IRES expression vector, the expression of the selection marker is made dependent on the successful transcription of the upstream gene of interest. As such, this reduces or eliminates the occurrence of selection marker expression without that of the gene of interest, which can occur due to gene fragmentation when a dual promoter dicistronic vector is used [33].This concept has been applied to improve the odds of picking a high producer cell clone even when gene amplification is used [29,34,35,36,37,38]. In a recent study, the application of IRES has also allowed for high recombinant protein production from MTX amplified cell pools, without the need for cloning [39].

### 2.2. Selection Marker Attenuation

When selection stringency is high [15], the surviving clones will have high transcript levels as a result of gene amplification or integration of the expression vector in a transcriptionally active spot in the genome, since the rate limiting step of recombinant protein expression occurs at the transcription process [40]. While selection stringency can be increased by increasing the drug concentrations in the cell culture, this approach is typically limited by the slower growth of cells when drug concentration very high. An alternative approach that has been explored is the attenuation of the selection marker. Theoretically, this allows higher selection stringency at lower drug concentrations since cells with low productivity of the selection marker gene will be selected against. Surviving cells are thereby forced to be more productive in the locus of the selection marker, thereby resulting in the high productivity of the adjacent gene of interest.

Two strategies have been employed to attenuate the selection marker. The first strategy involves the mutation of the selection maker to reduce its activity. This is demonstrated by the mutation of a selection marker, neomycin phosphotransferase II, to reduce its affinity to neomycin which leads to a subsequent improvement in the specific monoclonal antibody productivity of 1.4 to 14.6 fold [17] and 16.8 fold [31].The second strategy for selection marker attenuation is through the modulation of gene expression level of the selection marker. A variety of methods have been used to achieve the objective. Codon deoptimization of the selection marker gene through the use of least preferred codons of the expression host lowers the translation efficiency of the selection marker gene and hence leads to a reduction in protein expression [41]. Alternatively, the level of transcription of the selection marker gene can be moderated through the use of a weak Herpes simplex virus thymidine kinase (HSV-tf) promoter [16,42]. In addition, the use of AU-rich elements (ARE) and murine ornithine decarboxylase (MODC) PEST region as respective mRNA and protein destabilizing elements have been shown to successfully weaken the selection marker, which results in improvements in recombinant protein productivity using the MTX amplification system [42]. In a follow up study, an attenuated IRES element to reduce the expression of the downstream selection marker gene has also been employed to substitute ARE, resulting in high recombinant protein titers [39].

### 2.3. Matrix Attachment Regions (MARs)

MARs are genomic DNA sequences which serve as attachment points within the DNA that facilitate the anchoring of chromatin structure to the nuclear matrix during interphase [43]. Thus, MARs maintain a transcriptonally active chromatin structure through chromatin loops creation. Furthermore, MARs are associated with increased histone hyperacetylation which indirectly lead to demethylation of DNA to make it accessible to transcription machinery [44,45]. In addition to its chromatin modelling function, MARs also serve as binding sites for transcription factors like CCCTC binding factor (CTCF) and nuclear matrix proteins (NMP) to augment gene expression [46,47,48]. When used as cis acting elements or by flanking the transgene with MARs, the human β-globin MAR, chicken lysozyme MAR and β-interferon scaffold attachment region (SAR) have been shown to promote gene expression and increase the occurrence of high producing clones [46,49,50,51,52].

### 2.4. Ubiquitous Chromatin Opening Element (UCOE)

UCOE is an insulator element against heterochromatin expansion which is marketed by Merck Millipore [53]. It is a methylation-free CpG island that abolishes integration position dependent effects and maintains the chromatin in an “open” configuration to increase accessibility of the DNA region to transcription machinery [54]. Antibody production in CHO cells increased significantly upon the incorporation of UCOEs into expression vectors [53]. It has also been reported that UCOE increases the proportion of high producers and hence improving the expression of antibody by six fold in CHO stable transfection pools [55]. An alternative non-coding GC-rich DNA fragment is proposed to be a novel UCOE as flanking the gene of interest with the GC-rich fragment augments recombinant protein expression [56]. It was subsequently proposed that the rigidity of the GC bonds in a DNA double-helix allows the formation of DNA secondary structure that may affect methylation of DNA and histones which will influence the configuration of the chromatin [57,58,59].

### 2.5. Site-Specific Recombination

While traditional stable transfection strategies typically involve the random integration of foreign gene into chromosomes, site specific recombination offers an alternative strategy to develop high producing and stable clones in a reproducible and predictable manner [60]. This is made possible through the use of recombinases, which greatly improve the recombination efficiency in mammalian cell lines, in contrast to the low recombination efficiency of traditional homologous recombination. This method, commonly called site-specific recombination, requires the initial generation of a marked host cell line, prior to the introduction of the gene of interest and recombinase for targeted integration into the marked genomic site of the host cell line. To generate the marked host cell line, a reporter cassette flanked by short cis-acting DNA target sequences recognized by specific recombinases are randomly integrated into different loci in the genome via stable transfection. Subsequently, the transfected cell clones are screened for high expression of the reporter gene and single copy integration. Effectively, this will select for clones that have the reporter gene integrated into genomic loci which promotes high transcription rate of the reporter gene. The chance of the reporter gene integrating into these genomic loci (also known as genomic hot spots) is low as only 0.1% of the genomic DNA contains transcriptionally active sequences [61]. Nevertheless, once marked host cell lines that are high producers of the reporter gene are identified, a vector containing the gene of interest and the same or corresponding DNA target sequences, and a separate expression vector for the recombinase, are co-transfected into the marked host cell line. This leads to a strand recombination between the integrated reporter sequences and that of the gene of interest, thereby improving the odds of the gene of interest integrating into a genomic hot spot in the marked host cell line.

Two site specific tyrosine recombinases from the P1 phage and the yeast *Saccharomyces cerevisiae*, Cre and Flp respectively, are commonly used to recognize and recombine their respective short cis-acting DNA target sequences: 34 bp loxP sites and 48 bp Flp Recombination Target (FRT) [62,63,64,65,66,67,68]. The Cre/loxP system was first used for human monoclonal antibody production in CHO cells [69]. Recently, Kameyama *et al*. [70] artificially caused gene amplification through the use of multiple Cre-mediated integration process with mutated loxPs sequences to repeatedly insert multiple genes into one target site. Similarly, the Flp-In^TM^ cell line (Life Technologies, Carlsbad, CA, USA) was used for Flp mediated integration of 25 individual antibody expression cassette into specific FRT tagged sites in the genome to express human polyclonal anti RhD antibody [4]. Nevertheless, the reversibility of site specific recombination is a common drawback of the Cre and Flp recombinases as the recognition sites are recreated upon cassette exchange.

Another site-specific recombination technology makes use of integrase enzymes, such as λ integrase and φC31 integrase, which target 2 different sequences typically called attP and attB (attachment sites on the phage and bacteria respectively). Upon recognition of an attachment site previously integrated in the genome of the marked host cell, the integrase catalyze a recombination event which alters the attB and attP sites upon cassette exchange [71,72,73]. Since the integrase is unable to recognize the altered sites, the recombination is irreversible.

### 2.6. Artificial Chromosome Expression (ACE) System

The artificial chromosome expression (ACE) system consists of a mammalian based artificial chromosome known as Platform ACE, an ACE targeting vector (ATV) and a mutant λ integrase (ACE integrase) for targeted recombination [74,75]. Platform ACE consists of mainly tandem repeated ribosomal genes and repetitive satellite sequences which form the pericentromeric heterochromatin. It also has natural centromeres and telomeres to enable DNA replication without the need of integration into host cell genome, reducing the probability of chromosomal aberration and clonal heterogeneity. Due to higher proportion of AT base pairs to GC base pairs in the Platform ACE nucleotide composition, Platform ACE can be purified by high speed flow cell sorting [76] and subsequently be transfected to different cell types. Platform ACE is pre-engineered to contain 50–70 attP recombination acceptor sites, thus allowing the incorporation of multiple copies of the gene of interest. In a typical transfection, the Platform ACE cell line is cotransfected with the ATV and the ACE integrase plasmid. The recombination event activates the promoterless selection marker on the ATV by integrating the gene downstream of the SV40 promoter in the Platform ACE. Hence, cells that survive under application of respective selection pressure are identified as clones that have undergone correct recombination event. In addition, consecutive transfections using different selection markers can be carried out to saturate the recombination acceptor sites on the Platform ACE and hence, high copy number of gene of interest can be achieved without gene amplification.

Using this system, high expressing clones are reportedly selected from 100 to 200 cell clones, and monoclonal antibodies expressing cell line achieved yields greater than 500 mg/L in batch terminal shake flask cultures [74].

### 2.7. Cell Line Engineering

The quantity of recombinant protein expressed in a cell culture is dependent on the time integral of viable cell density (IVCD) and specific protein productivity (q) of the cells. To improve IVCD, cell line engineering strategies focus on extending the longevity of cell culture, accelerating the specific growth rate and increasing the maximum viable cell density [77,78,79,80,81]. Similarly, cell line engineering has been employed to improve the folding, transport and secretion of the recombinant protein to enhance q [82]. A variety of cell line engineering strategies which target diverse cellular functions of CHO cells such as apoptosis, autophagy, proliferation, regulation of cell cycle, protein folding, protein secretion and metabolites production IVCD have been comprehensively reviewed recently [2,83,84], and thus will not be covered in this review. Interestingly, cell engineering can also be used to simultaneously improve IVCD and q. For example, a combinatorial strategy of anti-apoptosis engineering and secretion engineering has produced a CHO cell line which expressed X-box binding protein 1 (XBP-1) and caspase-inhibitor, x-linked inhibitor of apoptosis (XIAP) [85]. XBP-1 is a potent transcription factor which binds to ER stress response element to stimulate promoters of the secretory pathway genes. This leads to an increase in overall protein synthesis which enhances q [86]. Nevertheless, it has been observed that expression of XBP-1 is correlated to reduced viability and stability of the engineered cell line. Thus, expression vector containing the XIAP gene was transfected into the cell line to inhibit apoptosis. Subsequently, over-expression of XIAP is shown to rescue the negative effects of XBP-1 on the cell line and it also leads to a 60% increase in titers.

Cell engineering effort is further boosted by the discovery of zinc finger proteins and transcription activator-like effectors (TALEs) which are protein domains that can be designed to recognize specific DNA sequences: Through varying the combination of the types and number of zinc finger proteins, DNA recognition modules that target unique sites (18–36 bp) on the genome can be created; As for TALEs, the DNA binding domain is a tandem array of repeating units, each of which targets one DNA base as determined by the amino acid residues at two specific positions in the highly conserved unit. [87,88,89,90]. By fusing the DNA-binding domain of these proteins to an endonuclease domain, zinc finger nucleases (ZFNs) and transcription activator-like effector nucleases (TALENs) can be created to target and cut specific DNA sequences [91]. The endonuclease domain of restriction enzyme Fok I has been used for this purpose, since it does not have a specific cleavage site and it requires dimerization to cleave DNA. Hence, a pair of ZFNs or TALENs targeting adjacent DNA sequences can position two of these Fok I endonuclease domains in proximity of each other to allow dimerization and thus DNA cleavage at the targeted DNA sequence. The resulting double stranded DNA break at the targeted gene loci is subsequently repaired by non-homologous end joining which often perturbs gene function. Alternatively, the co-transfection of a transgene with homologous region to the cut site can result in transgene integration at the nuclease targeted cleavage site because the presence of the double stranded break greatly stimulates homologous gene targeting via homologous end joining pathway. As the recognition module can be customized to target any DNA sequence, multiple genes can be targeted using this method to develop an optimized cell line. For example, a triple gene knockout [dihydrofolate reductase (DHFR), glutamine synthase (GS) and α1, 6-fucosyltransferase8 (FUT8)] CHO cells has been obtained through the use of ZFN [13]. The absence of DHFR and GS allows for selection of clones with high gene copy while the absence of FUT8 allows the production of mAbs with increased antibody dependent cell mediated cytotoxicity (ADCC) for higher treatment efficacy. Using the ZFN technology, a GS-knockout CHO cell line has also been generated and launched by Lonza. This is an improvement over the older GS system whereby a GS-containing CHOK1SV cell line is used. The absence of the GS gene in the new host cell line allowed for faster cell line development [92].

Another class of gene editing tools for targeted mutagenesis or transgene integration are meganucleases. These are sequence specific endonucleases that recognize DNA sites comprising of 12 or more base pairs. Due to their specificity, these enzymes are also used to create double strand break at targeted DNA site [93]. Meganuclease has also been applied to cell line development for targeted transgene integration which improves the efficiency in obtaining stably-expressing cell lines [83,94].

Improvement in genome-wide *in silico* modeling of mammalian systems has also identified novel pathway targets for modification in mammalian cell line [95,96]. Coupled with the availability of genome data and advancement of -omics tools, the field of mammalian cell line engineering has the potential to advance to an equivalent level of microbial cell line engineering. Thus, creation of optimized mammalian cell line through multiple genetic modifications to enhance stability and high expression of recombinant proteins is no longer a far-fetched concept.

## 3. Clone Screening Technologies

As a result of the random integration of foreign genes of interest and subsequent disruption of the genome by gene amplification systems, the cell clones obtained during cell line development are highly heterogeneous. Furthermore, high producing clones are typically rare in a population of transfected cells because the active region supporting high gene expression in the chromosome is rare [10] and these high producer cell clones typically have lower growth rates since a significant portion of resources are being used for expression of the recombinant protein [97]. Therefore, the screening of a large number of cell clones is commonly required to isolate the high producing clones.

**Figure 3:**
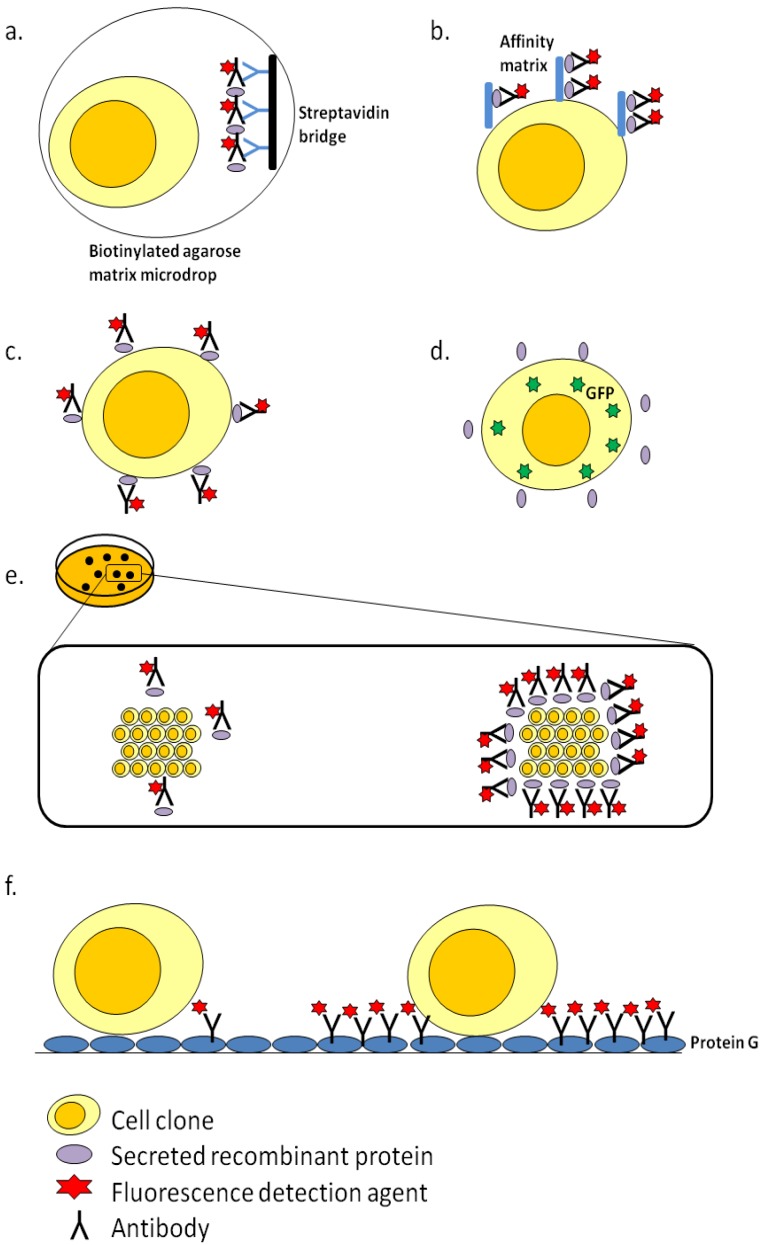
Fluorescence labeling strategies for different clone screening technologies to identify high producer cell clones. The coloring scheme for cell clone, primary antibodies, fluorescence agent and recombinant protein is yellow, black, red and purple respectively. All figures follow the same coloring scheme unless it is stated otherwise (**a**) Gel microdrop secretion assay used in FACS that encapsulate individual cells in biotinylated agarose matrix. Primary antibodies labeled with fluorescence agent binds to the recombinant protein and the complexes are subsequently bound to secondary antibodies (blue) immobilized on streptavidin bridge (black). (**b**) The affinity matrix attachment method used in FACS that cross linked the matrix to the cell surface within a gelatin based low permeability medium. Secreted recombinant protein remain bound to the affinity matrix (blue) which are subsequently probed by fluorescently labeled antibodies. (**c**) The cold capture method used in FACS where fluorescently labeled antibodies bind to secreted recombinant protein that remains associated with the cell surface at low temperature. (**d**) The expression of secreted recombinant protein is linked to an intracellular selection marker like green fluorescene protein (green) and the cell clones are processed by FACS. (**e**) In Clonepix system, cell clones are grown in semi solid media to limit the diffusion of the secreted recombinant protein. The secreted protein is captured by fluorescently labeled antibodies where they form a halo structure surrounding the cell colony. In this figure, the cell colony on the right is depicted as a high producer clone as compared to the cell colony on the left. After ranking by the Clonepix system, cells clones are transferred by micro-pins to a new well plate for further characterization. (**f**) For the Cell Xpress^TM^ system, cell clone expressing recombinant protein such as therapeutic antibodies (black) are captured by protein G (blue) in the well. The captured antibodies will be subsequently probed by fluorescence detection agent and screened by the system. In the figure, a high producer cell clone (right) and low producer cell clone (left) are shown. After ranking the cell clones in the same well, all cell clones except the highest ranking clone will be subjected to photomechanical lysis.

Traditionally, serial limiting dilution method is most commonly performed to screen for high producer cell clones due to its simple operation, despite being time, labour and capital intensive. In this method, cells are sequentially diluted on well-plates to obtain dilutions at which a portion of wells are devoid of cells. At the dilution, the wells containing cells will have expanded from a small subset of clones from the original cell pool. To ensure monoclonality, multiple rounds of serial subcloning steps is thus necessary, [98]. More importantly, additional steps of cultivating the cells and protein tittering typically by enzyme-linked immunosorbent assay (ELISA) are necessary to determine the protein productivity of the clones. Advancement in clone screening technologies can reduce the time and effort in this endeavour to find rare high-producing cell clones. Three such technologies are discussed in this section (Figure 3).

### 3.1. Fluorescence-Activated Cell Sorting (FACS)-Based Screening

FACS sorters are equipment that can simultaneously monitor the levels of multiple fluorescence wavelengths associated with a cell at a rate of 10^8^ per hour [99]. Cells to be analysed enter the FACS sorter singularly as a moving focused stream and they are interrogated by one or more laser beams. The resulting fluorescence from the cell is measured by relevant optical detector and the collected data is quantified and analysed. The machine then applies a charge to the droplet containing the cell to sort it into specific collection tube or well-plates. Depending on the fluorescence signal, cell parameters such as granularity and cell size can also be obtained [100]. However, accuracy of the FACS-based screening of high producer cell clones is dependent on the fluorescence signal that remains associated with the cell. Hence, it is more suited for selection of high producing cell clones that do not secrete its recombinant protein [101,102].

Nevertheless, several strategies have been proposed to select high producer cell clones that secrete its recombinant protein. The first strategy is known as the gel microdrop secretion assay which encapsulates the cell in a biotinylated agarose matrix with a diameter of 35 µm [103,104,105]. Fluorescently labeled primary antibody is added to the microdrop to bind to the secreted recombinant protein. Subsequently, secondary biotinylated antibody is used to capture the primary antibody and the complex will bind to a streptavidin bridge immobilized on the biotinylated agarose matrix. Hence the cell and its secreted protein remain in the microdrop for subsequent processing by FACS. The second strategy involves the cross linking of an affinity matrix on the cell surface within a gelatin based low permeability medium to capture the secreted recombinant protein [106,107]. Subsequently, the immobilized recombinant proteins are detected with fluorescent labeled antibodies and processed with FACS. While the two strategies have improved the ability of the FACS method in identifying and isolating high producers, the protocols for immobilizing the secreted recombinant proteins are often technically challenging and time consuming [108]. Consequentially, it was hypothesized that the amount of secreted recombinant protein which associates transiently with the cell surface should correlate with the total amount of protein being secreted from the cell clone. As such, a cold capture method was proposed. This involved the use of fluorescently tagged antibodies that binds to surface associated recombinant protein at low temperatures of 0–4 °C. Subsequently, the cell clones were subjected to three rounds of reiterative sorting by FACS to isolate the high producer clones. Using this method, clones with 20 fold increase in specific productivity as compared to the unsorted cell population were isolated [108].

Alternatively, strategies that measure the level of intracellular selection marker has been proposed as an indirect screen for high producing cell clones that secrete its recombinant protein. In one study, fluorescein isothiocyanate-labeled methotrexate (F-MTX) was used to bind to intracellular dihydrofolate reductase (DHFR) selection marker. The study demonstrated that the distribution of high producer cell clones is highest at a median level of F-MTX fluorescence intensity [102]. In another study, green fluorescent protein (GFP) was used as a second selection marker, and the correlation of GFP fluorescence and recombinant protein productivity is demonstrated with a correlation coefficient range of 0.52 to 0.70 [101]. Consequentially, depending on the intensity of fluorescence signal in the heterogeneous cell pool, FACS will be carried out to isolate high producer cell clones.

### 3.2. ClonePix

ClonePix FL system (Genetix, Sunnyvale, CA, USA) is an automated colony picker which is capable of screening large number of clones and identifying high producer clones within a short period of time. The process of ClonePix FL is initiated by cultivating single cells in a semi-solid media to allow the formation of individual colonies. The high viscosity of the media allows the progeny of the single cell to remain as a single colony and also trap the secreted recombinant proteins in close proximity to the secreting colonies. These secreted recombinant proteins are captured by fluorescein isothiocyanante (FITC) conjugated antibodies that were previously added to the semi-solid media. Upon capturing of the expressed recombinant proteins by the antibodies, they will be deposited as immunoprecipitates around the secreting cell clone and hence, forming a halo fluorescence structure [109]. Consequentially, clones are ranked according to the fluorescent intensity of the halo structure. High-ranking high producer cell clones are then aspirated by micro-pins and transferred to a new well plate for further characterization. The entire process of imaging 10,000 cell clones and selection of high producer cell clones is completed within an hour and it is sensitive enough to isolate rare high producing clones which formed 0.003% of the population [110]. Due to the high throughput nature of this method, the ClonePix FL system has been used in different studies to consistently select high producer cell clones expressing a range of recombinant proteins like green fluorescent protein and humanized antibody [73,111,112,113].

### 3.3. Cell Xpress^TM^

Cell Xpress^TM^ technology consists of the Cell Xpress^TM^ software module and the Laser-Enabled Analysis and Processing (LEAP^TM^) platform which are based on the principle of laser-mediated semiconductor manufacturing technologies. An advantage of the LEAP^TM^ platform is that it is built for high throughput operation and hence the entire screening process is fully automated and accomplished by robotics. The Cell Xpress^TM^ technology combines live cell imaging and laser-mediated cell manipulation to identify and purify the highest producer cell clone in a sample well [114]. Multi colour live cell imaging of Cell Xpress^TM^ technology is achieved through the simultaneous use of fluorescence detection reagents that associate specifically with either the cell clones or expressed recombinant protein. Furthermore, the sample wells are coated with capture matrix to mediate *in situ* capture of expressed recombinant protein as most of the recombinant proteins are secreted out of the cell. For example, if therapeutic antibodies are expressed, protein G is commonly used to capture the secreted antibodies. Typically, within the same well, custom software algorithms will locate the cell clones and create a kernel surrounding individual cell. The area of the kernel will then be expanded to include the secreted recombinant protein until adjacent kernels are encountered. Through measurement of the fluorescent intensity of the respective detection reagents, each cell in the same well will be ranked based on the amount of secreted recombinant protein. Additional criteria like cell growth rate, cell area and proximity to other cells will also affect the ranking. Hence, the highest ranking cell in a well will be identified as the most suitable high producer cell clone. Subsequently, laser mediated cell purification will commence whereby the laser beam is directed to the lower ranking cell clones in the same well via large field-of-view optics and galvanometer steering to induce photomechanical lysis [115]. High producing cell which remains in the well will be allowed to grow and finally be transferred to a larger well for expansion.

Since the purification process is a closed system, the probability of contamination is reduced. Cells obtained from a sample size range of 10 to 10^8^ cells have commonly reached 99.5% purity [115]. Furthermore, as the entire process requires less than 30 s to screen a single well, large numbers of cell clones can be screened within a short time to identify high producers. It has also been reported that the analysis result from Cell Xpress^TM^ technology shares a good correlation (R^2^ = 0.84) with the peak IgG volumetric productivities in shake flask growth and expression experiments [116]. In general, Cell Xpress^TM^ technology has routinely picked cell lines with specific antibody secretion rates of >50pg/cell per day [114]. Nevertheless, it has been reported that the laser used in LEAP^TM^ may damage high producing cell clone [10].

## 4. Conclusions

Besides its application in the expression of recombinant protein therapeutics, cell line development technologies are also critical in the expression of enzymes like dipeptidyl peptidase I (DPPI) which may expedite the industrial use of the peptidase as a processing enzyme [117]. Interestingly, these technologies may also be utilized for discovery and characterization of proteins that may be potential drug targets. This is because functional and structural studies of proteins may be limited by the quantity of biologically active protein expressed by current protein production approaches. While transient gene expression technologies may be sufficient in expressing these proteins, an alternative technology originally intended for generation of a stable cell line like the FRT/ Flp-In^TM^ recombinase system has been employed successfully to express a tumour-associated antigen, HAb18G/CD147, for functional studies [118]. Thus, advancement in new stable technologies that increases the speed and efficiency of generating high expressing stable cell clones may eventually replace transient gene expression technologies.

Going forward, the probability of breakthroughs in cell line development technologies is boosted by the availability of the CHO genome [119]. With this information, the use of comparative transcriptome analysis using completed CHO cell DNA microarray is facilitated, whereas research had been performed using incomplete CHO cell microarray [120,121] or non CHO derived DNA arrays [122,123] prior to the availability of CHO genomic information. In a recent study, data from large scale proteomic analysis was complemented by the genome data of CHO cells to discover a total of 6164 grouped proteins, which is an 8 fold increase over the identified proteins in the CHO cells proteome. Furthermore, the codon bias of CHO cells, which is distinct from human, was solved. These data will facilitate the expression of human proteins in CHO cells in future [124]. With the availability of techniques in the analysis of metabolites in CHO cells [125,126,127], combined data from genomics, transcriptomics, proteomics and metabolomics can identify novel genes that affect the growth and protein production rate of CHO cells.

Bioinformatics analysis is also boosted by the availability of the CHO genome. For example, the genomic data of CHO cells has facilitated *in silico* identification of CHO microRNA (miRNA) loci [128]. Thus, the profiling and subsequent use of microRNA (miRNA) to regulate gene expression has also increased in CHO cell line development in recent years [129,130,131,132]. This is because miRNA can be easily introduced into the cells and it can efficiently regulate multiple gene targets through mRNA cleavage or translation repression by interaction with 3' untranslated region (UTR) of the mRNA [133]. Furthermore, as miRNAs are non-coding RNAs, they present no additional translational burden to the cells. Alternatively, bioinformatics analysis of the CHO genome may also reveal new genomic hot spots for site-specific integration of the gene of interest to generate high producing clones. This has been previously accomplished in human genome [46].

Besides the focus on the increased production of protein therapeutics, there will also be a need to improve the quality of the recombinant protein product which entails metabolic engineering of CHO cells to perform post translational protein modification. For example, the N-acetylglucosaminyltransferase-III gene has been overexpressed in CHO cells to ensure accurate protein glycosylation pattern in protein therapeutics [134]. Taking it further, there has been an attempt to produce non protein therapeutic like heparin in CHO cells [135]. While the composition of disaccharide species from expressed heparin sulfate differs from pharmaceutical heparin in the study, it was proposed that fine tuning the expression of transgenes involved in heparin synthesis pathway may solve the problem. In conclusion with the new technologies discussed above, new tools in cell line development can be generated and the process can be further streamlined to facilitate biopharmaceutical drug discovery and development.
